# Is Future Mental Imagery Associated with Reduced Impact of the COVID-19 Pandemic on Negative Affect and Anhedonic Symptoms in Young People?

**DOI:** 10.1007/s10608-023-10352-1

**Published:** 2023-02-04

**Authors:** Taryn Hutchinson, Laura Riddleston, Victoria Pile, Alan Meehan, Meenakshi Shukla, Jennifer Lau

**Affiliations:** 1 Department of Psychology, Institute of Psychiatry, Psychology & Neuroscience, King’s College London, London, UK; 2 Department of Psychology, University of Allahabad, Prayagraj, India; 3 Youth Resilience Unit, Wolfson Institute of Population Health, Queen Mary University of London, London, UK

**Keywords:** Adolescence, Depression, Anhedonia, Mental imagery, COVID-19 stress

## Abstract

**Background:**

Difficulties with prospective mental images are associated with adolescent depression. Current treatments mainly focus on verbal techniques to reduce negative affect (e.g. low mood) rather than enhancing positive affect, despite anhedonia being present in adolescents. We investigated the concurrent relationships between the vividness of negative and positive prospective mental imagery and negative affect and positive affect; and examined whether negative and positive prospective mental imagery moderated the impact of recent stress (COVID-19-linked stress) on negative and positive affect.

**Methods:**

2602 young people (12–25 years) completed the Prospective Imagery Task and self-reported on symptoms of negative affect, anhedonia and COVID-19 linked stress.

**Results:**

Elevated vividness of negative future mental imagery and reduced vividness of positive future mental imagery were associated with increased negative affect, whereas only reduced vividness of positive future imagery was associated with increased symptoms of anhedonia. Elevated vividness of negative future images amplified the association between COVID-19 linked stress and negative affect, while elevated vividness of positive future images attenuated the association between COVID-19 linked stress and anhedonia.

**Conclusions:**

Future mental imagery may be differentially associated with negative and positive affect, but this needs to be replicated in clinical populations to support novel adolescent psychological treatments.

**Supplementary Information:**

The online version contains supplementary material available at 10.1007/s10608-023-10352-1.

## Introduction

Depression is common in adolescents and young people (Zhou et al., 2020), with 2.7% of 11–16 year olds and 4.8% of 17–19 year olds, in the UK (Mental Health of Children & Young People in England, 2017 [PAS], 2018) and 1.1% of 10–14 year olds and 2.8% of 15–19 year olds, globally (World Health Organization [WHO], 2021), experiencing an episode of major depressive disorder. Youth depression is associated with disruptions to education, socialising and increased risk of developing long-term physical and/or mental health problems (Maughan et al., 2013; Thapar et al., 2012). Yet, current treatments for adolescent depression are suboptimal (Lewandowski et al., 2013). One explanation is that frontline psychological interventions primarily use verbal techniques focussed on the present moment (“here and now”) (Westbrook et al., 2007). Disruptions in prospective mental imagery plays a key role in adolescent depression, yet it is commonly neglected in current interventions (Pile et al., 2021). A second limitation of current treatments is that they tend to focus solely on alleviating negative mood rather than enhancing positive affect, despite adolescent depression involving anhedonic symptoms (Clark & Watson, 1991; Craske et al., 2016; Dunn, 2012). Improving our understanding of key cognitive factors that might be differentially associated with positive vs negative affect could enable more precise matching of specific difficulties to treatment techniques, enhancing current treatments. Here, we investigated: (a) the concurrent relationships between the vividness of positive and negative prospective mental imagery and positive and negative affect, and (b) whether these aspects of future imagery moderated the impact of stress—those posed by the uncertainty and lockdown restrictions of the coronavirus pandemic (COVID-19)—on negative and positive affect. In our large sample of 2602 young people, we also confirmed the factor structure of the Prospective Imagery Task (PIT) and explored the effects of demographic variables (age, sex and ethnicity on aspects of future imagery).

Mental imagery is described as representations of sensory information without external input (Pearson et al., 2015). Unhelpful *emotional* mental imagery (including frequent, vivid and distressing images of past events and those of future goals) may play a key role in the development and maintenance of mood disorders. To date, most research has focussed on distressing (negative) imagery of past events, with between 44 and 87% of depressed adults reporting frequent vivid, distressing, and unwanted memories of significant life events which are associated with negative emotions, including dysphoria concurrently (Birrer et al., 2007; Brewin et al., 1996, 2009; Kuyken & Brewin, 1994; Patel et al., 2007), and prospectively, even after controlling for early symptoms (Brewin et al., 1999). Distressing and intrusive images of the past may also maintain depressive mood in young people (Kuyken & Howell, 2006; Meiser-Stedman et al., 2012). Negative memories are more vivid in depressed adolescents than those who have never been depressed (Kuyken & Howell, 2006) and increased frequency of intrusive images, which may relate to vivid negative memories, are linked with higher symptoms of depression in adolescents (Meiser-Stedman et al., 2012). Targeting these distressing images in treatments—for example, through imagery rescripting—can reduce depression (and anxiety) in adults (Brewin et al., 2009; Wheatley et al., 2007) and adolescents (Pile et al., 2021).

A more recent line of research has focussed on biases in *future* mental imagery (Roepke & Seligman, 2016). Whereas studies of mental imagery of past events focus mainly on negative images, future mental imagery, which underlies the ability to make predictions about the future, has been studied in relation to the forming of specific and detailed plans around positive outcomes e.g., goals (Boland et al., 2018; Szpunar et al., 2014). The ability to vividly imagine a goal-related future event may help individuals plan actions and anticipate potential barriers to work towards their desired goal (Taylor & Schneider, 1989). If depressed individuals have difficulty simulating vivid images of specific events and instead, generate more general future expectancies (Anderson et al., 2016), then they will also predict future goals as less likely to occur and being less controllable (Dickson et al., 2011), which in turn may drive feelings of hopelessness about the future (Beck, 1979; Rubenstein et al., 2016b). Consistent with this, research in adults has consistently shown that depression (rather than anxiety) is associated with impoverished future mental images of positive events (Anderson & Evans, 2015; Holmes et al., 2008b; MacLeod & Byrne, 1996; Morina et al., 2011). Within this body of work, experimental studies show that imagining positive events has a specific impact on positive affect over time in healthy and dysphoric individuals (Holmes et al., 2008a, 2008b). These effects are thought to translate into actual goal attainment, as generating positive imagery increases the vividness of positive images (Boland et al., 2018; Pictet et al., 2011) and the perception that these events are more important, controllable, and likely to occur in dysphoric individuals (Boland et al., 2018). In depressed individuals, positive future images also shape more behavioural activation (Renner et al., 2017).

Far fewer studies have explored the association between positive images of the future and the absence of positive affect in young people, despite the importance of these findings for treatment models of anhedonic symptoms, which could bridge the gap in efficacy of current treatments. To date, only one study to our knowledge has measured prospective mental imagery in relation to depression (and anxiety) symptoms in young people (Pile & Lau, 2018). This found that adolescents with high depression (rather than anxiety) scores were associated with less detailed, vivid images of future positive events. In contrast, both anxiety and depression symptoms associated with more detailed and vivid images of past and future negative events. Thus, the absence of positive affect that uniquely characterises depression associates with deficits in future positive imagery while negative affect that characterises depression and anxiety associates with deficits in future negative images, but these specific associations need validation.

This study advances existing knowledge by exploring associations between positive and negative prospective images with negative affect and the absence of positive affect (anhedonia) in a large sample of adolescents and young adults across a broad age range (12–25 years). We aimed to test the primary hypotheses that reduced vividness of positive future images would be associated with increased symptoms of anhedonia in young people while elevated vividness of negative future images would be associated with increased negative affect. As past studies have found that cognitive factors, such as attributional style can moderate the impact of negative life events on symptoms of depression in adults (Rubenstein et al., 2016a), adolescents (Joiner & Wagner, 1995; Lakdawalla et al., 2007), and children (Vines & Nixon, 2009), we also aimed to assess whether future imagery variables moderated the effects of stress on symptoms of depression. A previous study showed that vividness of positive imagery moderated the relationship between the severity of negative life events and symptoms of depression in adolescents (Pile & Lau, 2018) and here we extended that finding to distinct symptoms of depression (negative affect, anhedonia). We tested these predictions on anhedonia and negative affect that emerged during the COVID-19 pandemic, measuring individual differences in self-reported stress. The COVID-19 pandemic has been a period of high disruption to the lives of young people globally, through school closures, social isolation, and quarantine (Brookes et al., 2020; Lee, 2020; Loades et al., 2020) and symptoms of depression have significantly increased from before to during the pandemic in adolescents aged between 9 and 18 years in the USA, Netherlands, Peru, and Australia (Barendse et al., 2022; Magson et al., 2021). Finally, whilst the Prospective Imagery Task (PIT) has been adapted for use in young people, previous studies have not had large enough samples to explore its factor structure. Therefore, we also aimed to explore the factor structure of the PIT in young people, as well as the effects of demographic factors (age, sex, ethnicity minority status and parental educational levels) on mental imagery variables. Understanding the cognitive mechanisms of positive and negative future imagery in relation to anhedonia and negative affect may provide useful insights for treatment of adolescent depression.

## Methods

### Materials and Methods

#### Participants and Procedures

Participants were 2602 young people aged 12–25 years (mean: 18.42 years; SD = 3.59; 70.3% female; 49.9% White) recruited through an online survey conducted from the UK. Although participants were recruited through educational institutions, charities and voluntary organisations based in the UK, social media platforms (which nonetheless were registered through UK-based users or organisations) were also used for recruitment, and a minority of participants (n = 42, 1.61%) accessed the survey from countries outside of the UK and reported living elsewhere (see supplementary materials for details). These “overseas” participants were significantly older (mean: 21.05 years; SD = 2.73), *F*(1,2600) = 8.61, *p* < .001, but the proportion of female to male participants was not significantly different to the UK sample, *F*(1,2600) = 24.34, *p* = .062.

The study received ethical approval from the Psychiatry, Nursing and Midwifery Research Ethics Committee at King’s College London (ref: HR-19/20-18250). An online survey design was used to understand how young people were managing their emotions across time during the COVID-19 pandemic. Data was collected using the online platform, Qualtrics. Data collection started on 12th May 2020 and finished on 1st December 2020; participants were given the opportunity to complete up to 8 surveys in total, one survey every 2 weeks for up to 16 weeks. Participants who completed the first 4 surveys were reimbursed with a £10 voucher and those who completed the final 4 surveys received a second £10 voucher. 4872 responses were recorded at the baseline survey and 2270 responses were removed due to the following criteria: (1) not answering any survey questions; (2) duplicate responses; (3) not meeting inclusion criteria (outside of age range); (4) minimum survey completion time was lower than 5 min; inauthentic responses identified by pre-set criteria. 900 participants completed all 8 surveys and the mean number of surveys competed was 4.62 (see supplementary materials for average length of time between surveys). In this study, we chose to focus on only the baseline data to demonstrate the validity of using the PIT in young people across a wide age range (12–25 years) and also that our data confirmed the expected factor structure.

Upon clicking the survey link, participants were presented with information about the study and, depending on which country they were residing in at the time and their age, provided consent for themselves or were instructed how to secure parental consent. After providing consent, participants completed a series of questions about their age, sex, ethnicity, parental educational qualifications, current education level and school attendance. They also completed questions around the impact of the COVID-19 pandemic on different life domains, before completing questionnaires that assessed negative affect, anhedonia, well-being, loneliness and boredom, along with measures of prospective mental imagery, attention control and appraisal abilities. They were also asked to complete open-ended questions on their worries, and how they managed negative emotions and loneliness. The present analysis focuses only on measures of negative affect, anhedonia and prospective mental imagery.

#### Measures

##### Demographic Characteristics

Participants provided their sex assigned at birth and the month and year of their birth. Ethnicity information was collected using questions based on the Office of National Statistics (ONS) recommendations (ONS, 2017). Socio-economic status (SES) was measured by asking participants their parents’ highest academic qualification.

##### Positive and Negative Affect Schedule (PANAS)

The Negative subscale of the PANAS (PANAS-N) (Watson et al., 1988) was used to measure negative affect over the previous 2 weeks. The PANAS-N consists of 10 items, rated on a 5-point Likert scale, with higher scores indicating greater negative affect. The PANAS-N demonstrated good internal consistency (Cronbach’s α = .87), which is similar to a previous study that explored the internal consistency of the PANAS-N in a large adolescent sample; (Cronbach’s α = .88) (Ortuño-Sierra et al., 2019). The PANAS has convergent and discriminant validity with measures of anxiety, depression and personal well-being in young people (Ortuño-Sierra et al., 2019; Sandín, 2003).

##### Snaith-Hamilton Pleasure Scale (SHAPS)

The SHAPS (based on Snaith et al., 1995) was adapted for use in young people during the pandemic by removing five items (see supplementary materials). It is a measure of anhedonia, and nine statements are presented to participants (e.g., “I will enjoy my favourite television or radio programme”). Participants indicated how much they agreed or disagreed with each statement on a 4-point Likert scale (Franken et al., 2007). Higher scores indicate greater anhedonia. The internal consistency for the SHAPS was acceptable (Cronbach’s α = .79), though a previous study with a large adolescent sample found the SHAPS to have good internal consistency (Cronbach’s α = .87) with convergent and discriminant validity (Leventhal et al., 2015).

##### The Prospective Imagery Task (PIT)

The PIT is a measure of vividness for prospective mental imagery and was originally used with adults (Holmes et al., 2008a; Stöber, 2000) but has been adapted for use in young people (Pile & Lau, 2018, 2020). Fourteen scenarios (7 positive and 7 negative) were presented to participants, with some scenarios containing more general events (e.g., “You will achieve something you wanted to”) and others were more specific (e.g., “You will have a serious argument with a friend”). Participants were asked to imagine each happening to them and then rate the vividness of this mental image on a 5-point scale (from ‘No image at all’ to ‘Very clear and detailed’). Higher scores indicate more vivid future images on the two scales. The positive scale demonstrated good internal consistency (Cronbach’s α = .83) and acceptable internal consistency was noted for the negative scale (α = .75).

##### Impact of COVID-19

This is an 8-item measure of stress associated with COVID-19 (and associated lockdown measures) on daily routines, including work, study, finances, social life (including leisure activities), relationships with family, physical health, emotions and caring responsibilities (for children/siblings or elderly/fragile family members). Participants were asked to rate how much they felt the coronavirus pandemic impacted each of these areas on a 6-point Likert scale (1 = not applicable, 6 = severely), over the last 2 weeks. Responses were summed to create a total impact score. The internal consistency for the impact items was questionable (Cronbach’s α = .63).

### Data Analysis

The first set of analysis focused on confirming the 2-factor structure of the Prospective Imagery Task and investigating whether there were any effects of demographic factors (age, sex, ethnicity, SES) on emotional mental imagery. For ethnicity, we compared those who were white versus minority ethnic status, and for SES, we compared presence versus absence of a parental degree. Using MPlus v8 (Muthén & Muthén, 2017), confirmatory factor analysis (CFA) was conducted to assess the construct validity of the PIT, using a two-factor structure for positive future imagery and negative future imagery. Of note, we also assessed whether a one-factor structure fit the Impact of COVID-19 data. Model fit was assessed using goodness-of-fit measures that are recommended for large datasets: (a) the root mean square error of approximation (RMSEA; good fit < .05, acceptable fit < .08); (b) standardised root mean square residual (SRMR; good fit < .08); and (c) the comparative fit index [CFI; good fit > .95, acceptable fit > .90)] (Hooper et al., 2008; Hu & Bentler, 1999; MacCallum et al., 1996; Pickard et al., 2018). To assess demographic effects on vividness of positive or negative future mental imagery, we performed a 2 × 2x2 × 2 mixed-measures ANCOVA with valence (positive, negative) as a within-subject factor; sex (male, female), ethnicity (white, minority groups) and SES (presence, absence of a parental degree) as between-subject factors; and age as a continuous covariate. For any main effects and interactions, independent samples t-tests were performed to see if any differences between groups were statistically significant, and if so, what the effect sizes were.

**Fig. 1:**
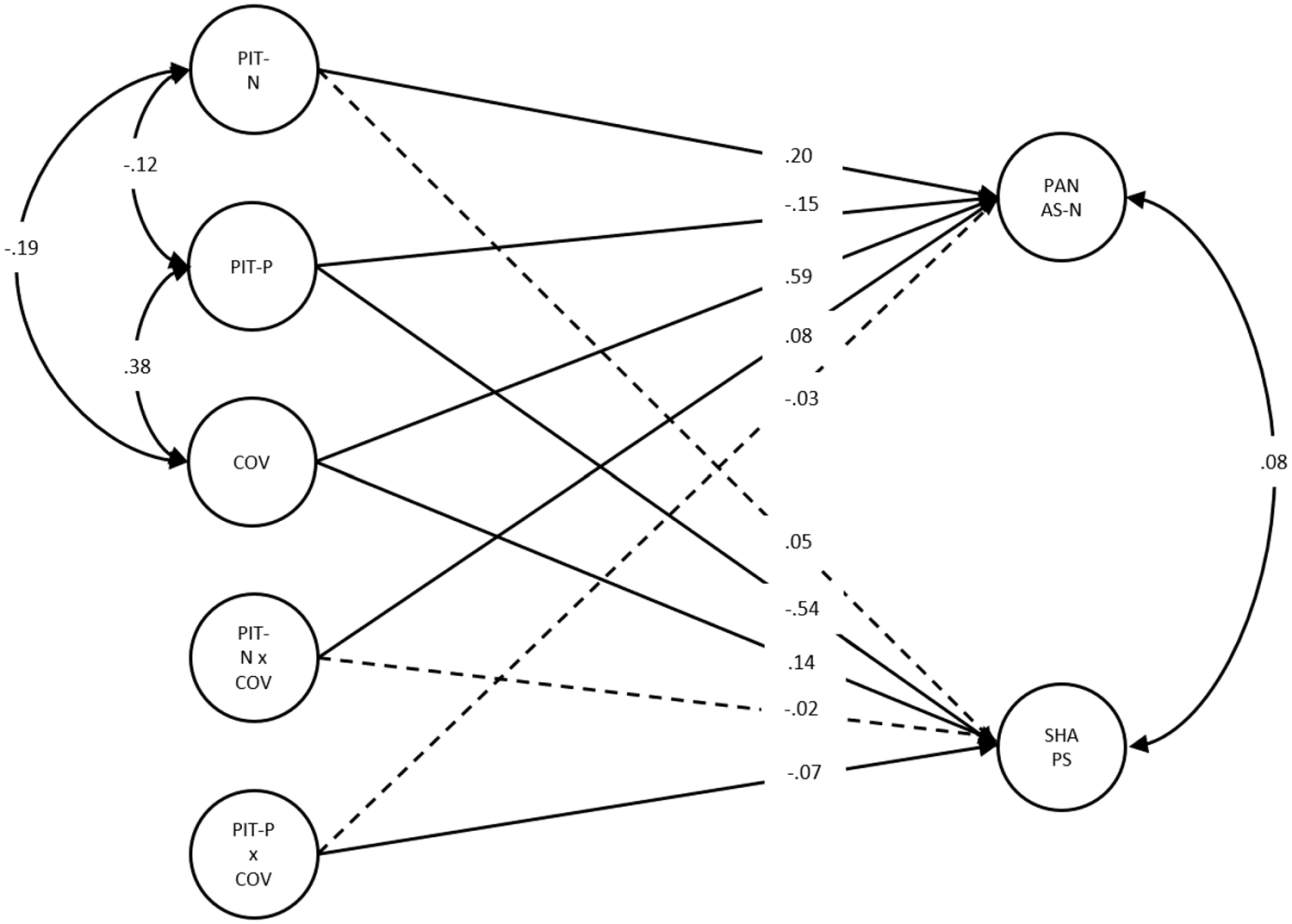
Moderation SEM model testing whether positive future imagery and negative future imagery moderate the effect of the impact of COVID-19 on negative affect and anhedonia symptoms. *Note.* Standardised beta coefficients and standard errors are reported. All analyses controlled for age, sex, SES and ethnicity. Solid lines indicate significant associations at *p* < .05

**Fig. 2:**
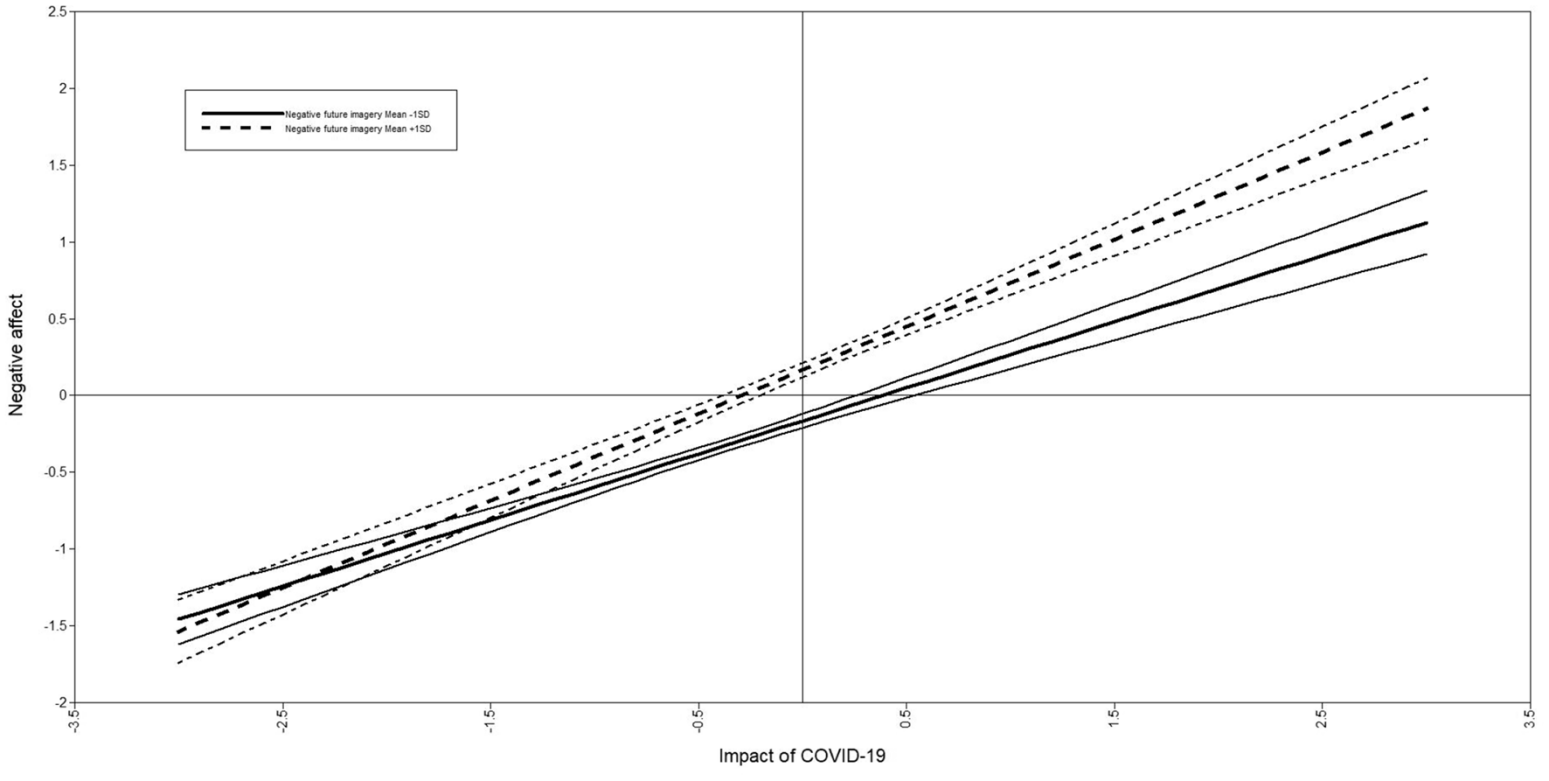
Simple slopes graph for the moderation effect of negative future imagery on the relationship between the of impact of COVID-19 and negative affect. *Note.* Solid lines indicate low negative future imagery (1SD below the mean), and broken lines indicate high negative future imagery (1SD above the mean). Thinner lines indicate 95% confidence intervals

**Fig. 3:**
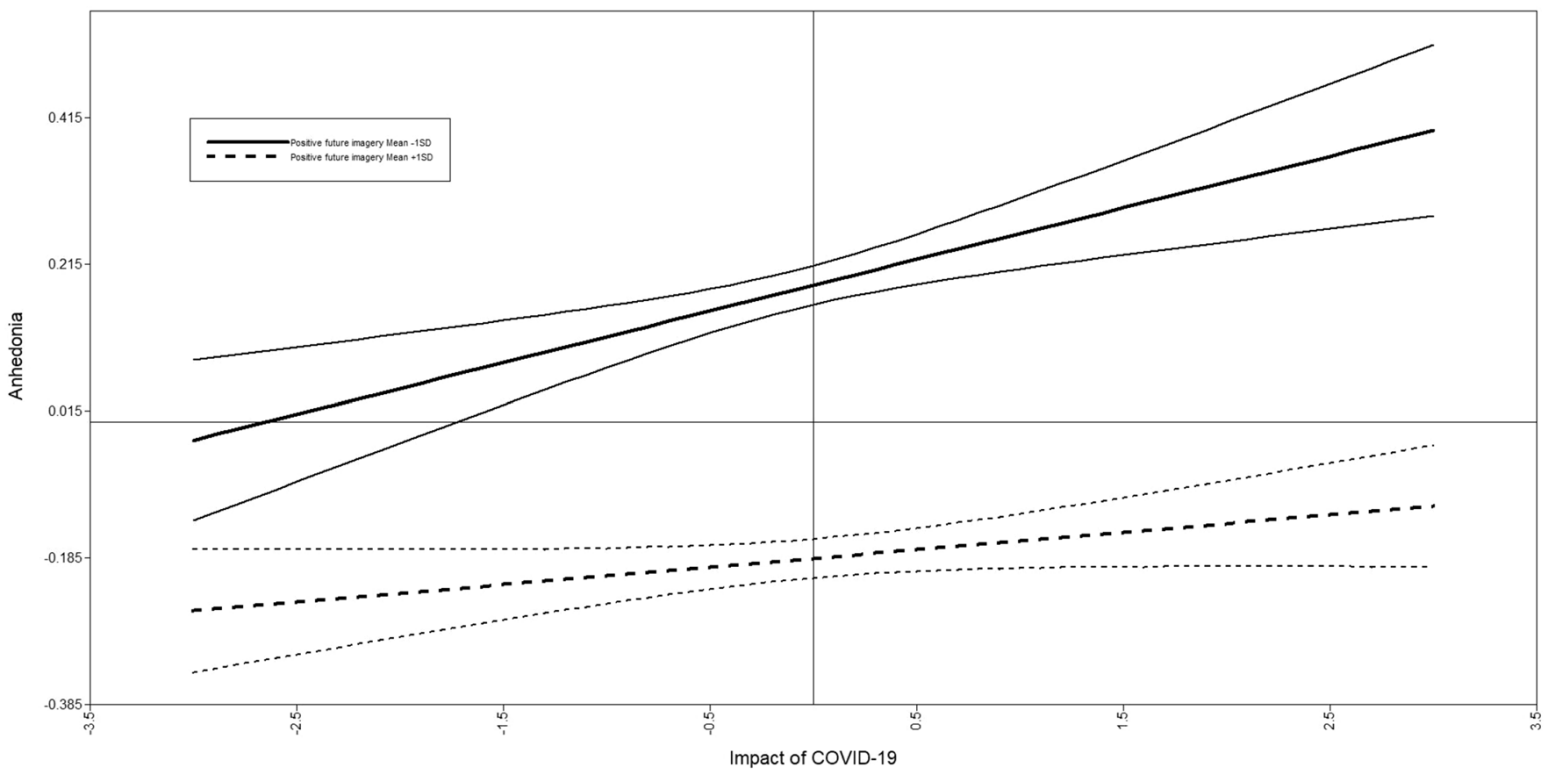
Simple slopes graph for the moderation effect of positive future imagery on the relationship between the of impact of COVID-19 and anhedonia. *Note.* Solid lines indicate low positive future imagery (1SD below the mean), and broken lines indicate high positive future imagery (1SD above the mean). Thinner lines indicate 95% confidence intervals

The second set of analyses focused on our specific hypotheses. We first explored the relationships between vividness of positive and negative future mental imagery and negative affect and anhedonia through correlations. Then, we conducted structural equational modelling (SEM) in MPlus to assess the associations between *predictor variables* (negative future imagery, positive future imagery and the impact of COVID-19), and the interactions between them on the *outcome variables* of negative affect and anhedonia (Fig. 1). SEM was chosen over regression analyses, as SEM permitted us to test our hypotheses more closely by allowing us to simultaneously test complex relationships between several different variables, as well as allowing us to define and compare latent variables whilst minimising the measure error that would have otherwise been included if we had simply created a single total score. SEM also allowed us to test the fit of the model to our data. All relationships were tested between latent factors, which are factors that cannot be directly measured but can be inferred through measuring related variables (Field, 2000). As latent factors are free from measurement error, we were able to capture more robust constructs (Field, 2000; Pickard et al., 2018). Each latent factor was specified within the model, for example the 10 items of the PANAS-N loading onto the negative affect latent construct (see supplementary materials for details). All latent factors were free to covary, and all paths were reported as partial regression coefficients. Age, sex, SES and ethnicity were included as covariates. Model fit was assessed using the Akaike (AIC) and Bayesian (BIC) criteria. Model parameters were estimated using maximum likelihood estimation with a robust sandwich estimator and the Yuan and Bentler (2000) test statistic. Missing data was accounted for using full information maximum likelihood. To interpret the moderation effects, we examined the simple slopes for high (1 SD above the mean) and low (1 SD below the mean) levels of negative future imagery on the relationship between the impact of COVID-19 and negative affect (Fig. 2); and high (1 SD+) and low (1 SD-) levels of positive future imagery on the relationship between the impact of COVID-19 and anhedonia (Fig. 3).

## Results

Table 1 shows the means and standard deviations for negative affect, anhedonia, positive and negative future imagery, and the impact of COVID-19 variables for the whole sample and by sex.

**Table 1:** Means and standard deviations for negative affect, anhedonia, positive and negative future imagery, and the impact of COVID-19 for the whole sample and by sex

	Whole sample mean (SD)	Female mean (SD)	Male mean (SD)
	N = 2602	N = 1828	N = 774
Negative affect	23.12 (8.10)	24.23 (8.01)	20.48 (7.70)
Anhedonia	16.35 (4.15)	16.28 (4.14)	16.52 (4.19)
Positive future imagery	24.48 (5.34)	24.12 (5.31)	25.34 (5.30)
Negative future imagery	22.55 (5.43)	22.91 (5.35)	21.69 (5.51)
Impact of COVID-19	30.81 (6.16)	31.31 (6.01)	29.64 (6.34)

### Confirmatory Factor Analysis

All fit indices for the two-factor model of the PIT (RMSEA [CI] = .055 [0.05, 0.06], CFI = .923, SRMR = .039) were indicative of acceptable-to-good model fit. The results imply that the PIT coherently measures two distinct constructs, including positive future imagery and negative future imagery (Table 2). All fit indices for the one-factor model of the Impact of COVID-19 scale (RMSEA [CI] = .076 [0.068, 0.083], CFI = .870, SRMR = .044) were indicative of an acceptable model fit, although the CFI was slightly below this threshold. The results imply that the Impact of COVID-19 scale measures one single construct.

**Table 2:** Factor loadings based on confirmatory factor analysis with orthogonal rotation for the 14 items of the Prospective Imagery Task (PIT) (N = 2424)

	PIT-N	PIT-P
You will have a serious argument with a friend	0.553	
You will be unwell	0.563	
You will feel that people don’t understand you	0.667	
You will find your (school) work really difficult	0.553	
Things won’t work out as you had hoped	0.634	
People will find you dull and boring	0.690	
People won’t like you	0.757	
You will have lots of energy and enthusiasm		0.666
You will do well at school/ work		0.651
You will make good and lasting friendships		0.700
You will achieve something you wanted to		0.681
People you meet will like you		0.688
You will be very fit and healthy		0.634
You will have lots of fun with friends		0.682

### Vividness of Future Mental Imagery and Demographic Factors

A mixed measures ANCOVA was conducted with valence as a within-subjects factor, sex, ethnicity and SES as between-subject factors, and age as a continuous covariate. This revealed a significant within-subjects effect of mental imagery valence *F*(1,1984) = 32.20, *p* < .001 such that higher ratings were assigned to positive over negative events. There was only one significant between-subjects effect, that of SES, *F*(1,1984) = 8.40, *p* = .004, *d* = .*204*—driven by higher vividness rating (independent of valence) in those participants whose parent(s) had a degree (*M* = 23.77*, SD* = 3.55) compared to those who did not (*M* = 23.00, *SD* = 3.99). There was no interaction between valence and SES, *F*(1,1984) = 1.71, *p* = .191. However, there were significant interactions between: (a) valence and age, *F*(1,1984) = 10.41, *p* = .001; (b) valence and sex, *F*(1,1984) = 34.25, *p* < .001; and (c) valence and ethnicity, *F*(1,1984) = 8.86, *p* = .003. There were no 3-way interactions,* p* > .05, nor 4-way interactions, *p* > .15.

To decompose the interaction between valence and age, we looked at the correlation between age and each subscale. Vividness of positive future images was negatively correlated with age, *r* = − .086, *p* < .001 but the vividness of negative future imagery was not significantly correlated with age, *r* = .022, *p* = .264. The significant valence x sex interaction was explained by different patterns of sex difference on each subscale. Female participants (*M* = 24.12, *SD* = 5.32) showed significantly *reduced* vividness of positive future imagery compared to male participants (*M* = 25.34, *SD* = 5.30, *t*(2499) = 5.25, *p* < .001, *d* = .230). However, female participants ((*M* = 22.91, *SD* = 5.35) had significantly *greater* vividness than male participants (*M* = 21.69, *SD* = 5.51, *t*(2502) = -5.16*, p* < .001, *d* = − .226) on negative future imagery. To decompose the significant valence x ethnicity interaction, we compared minority status differences for each subscale. White participants (*M* = 24.22, *SD* = 5.20) had significantly reduced vividness of positive future imagery compared to minority groups participants (*M* = 24.71, *SD* = 5.36, *t*(1997) = − 2.00, *p* = .049, *d* = − .092). Vividness of negative future imagery was not significantly different between white (*M* = 22.79, *SD* = 5.29) and minority groups (*M* = 22.39, *SD* = 5.38, *t*(1999) = 1.62, *p* = .105, *d* = .075).

### Correlations Between Future Mental Imagery and Negative Affect and Anhedonia

To explore the relationship between the valence of future imagery and depressive symptoms, we looked at correlations between the vividness of each future imagery subscale and negative affect and anhedonia. Vividness ratings for negative future images were positively correlated with increased negative affect, *r* = .40, *p* < .*0*01, whereas ratings for positive future images were negatively correlated with negative affect, *r* = − .24, *p* < .001. Vividness of positive future images were negatively correlated with anhedonia, *r* = − .44, *p* < .001, and negative future images were positively correlated with anhedonia, *r* = .11, *p* < .001.

### Hypothesis-Testing of Associations Between Imagery Variables and Negative Affect and Anhedonia

Results from the SEM model (AIC = 232482.45, BIC = 233297.67) analyses revealed that both negative future imagery (β(se) = .20(.03), [95% CI] [0.14, 0.25], *p* < .001) *and* positive future imagery (β(se) = − .15(.02), [95% CI] [− .18, − .10], *p* < .001) were significantly associated with *negative affect*. Furthermore, negative future imagery significantly moderated the relationship between the impact of COVID-19 and negative affect (β(se) = .08(.02), [95% CI] [0.32, 0.44], *p* < .001), but positive future imagery did not (β(se) = − .03(.02), [95% CI] [− .25, − .13], *p* = .150). Using simple slopes analysis to interpret the significant interaction revealed that at low vividness of negative future imagery, there was a significant positive relationship between the impact of COVID-19 and negative affect, (*B*(se) = .43(.03), [95% CI] [0.37, 0.49], *p* < .001*)* and this relationship was strengthened with high vividness of negative future imagery, (*B*(se) = .57(.03), [95% CI] [0.50, 0.63] *p* < .001*)*. Finally, sex was the only covariate significantly associated with negative affect (β(se) = .10(.02), [95% CI] [0.06, 0.13], *p* < .001) (see supplement for the full moderation SEM model).

Positive future imagery (β(se) = − .54(.02), [95% CI] [− .22, − .15], p < .001) was significantly associated with *anhedonia*, but negative future imagery was not (β(se) = − .50(.03), [95% CI] [− .01, 0.05], p = .108). Positive future imagery significantly moderated the relationship between the impact of COVID-19 and anhedonia (β(se) = − .07(.03), [95% CI] [− .05, 0.00], p = .025), but negative future imagery did not (β(se) = − .02(.03), [95% CI] [− .03, 0.02], p = .502). Again, using simple slopes to interpret the significant interaction, we found that at low vividness of positive future imagery the relationship between the impact of COVID-19 and anhedonia was significant, (*B*(se) = .07(.02), [95% CI] [0.03, 0.11], *p* < .001), however at high vividness of positive future imagery, this relationship was no longer significant, (*B*(se) = .02(.01), [95% CI] [− .00, 0.05], *p* = .077). Age (β(se) = − .06(.01), [95% CI] [− .01, 0.00], p = .012) and sex (β(se) = − .08(.02), [95% CI] [− .11, − .02], p < .001) were the only covariates significantly associated with anhedonia.

In addition, there were significant main effects of the impact of COVID-19 on negative affect (β(se) = .59(.02), [95% CI] [0.43, 0.57], *p* < .001) and anhedonia (β(se) = .14(.03), [95% CI] [0.02, 0.08], *p* < .001). The impact of COVID-19 also significantly correlated with negative future imagery (β(se) = .38(.03), [95% CI] [0.31, 0.46], *p* < .001) and positive future imagery (β(se) = .19(.03), [95% CI] [− .27, = .13], *p* < .001). Finally, positive future imagery and negative future imagery were significantly negatively correlated (β(se) = − .12(.04), [95% CI] [− .21, − .02], *p* = .002).

## Discussion

This study investigated the differential relationships between vividness of negative and positive future imagery, and negative and positive affect in a large sample of adolescents and young adults. First, we found that elevated vividness of negative future mental imagery *and* reduced vividness of positive future mental imagery were associated with increased negative affect, whereas only reduced vividness of positive future imagery was associated with increased symptoms of anhedonia. Second, elevated vividness of negative future images amplified the association between the impact of COVID-19 and negative affect while elevated vividness of positive future images attenuated the association between the impact of COVID-19 and anhedonia. Finally, the Prospective Mental Imagery Task showed the expected two-factor structure in young people.

Whilst theoretical frameworks such as the Tripartite Model (Clarke & Watson, 1991) have identified shared and unique cognitive correlates between anxiety and depression—findings that appear to characterise adolescents (Pile & Lau, 2018)—our findings spoke to distinct links between future imagery variables and distinct dimensions of depression (negative affect and anhedonia). Our findings went further by suggesting that positive and negative future imagery also had differential moderating effects on the relationship between the impact of COVID-19 and negative affect and anhedonia. Those with more vivid negative future images showed a stronger association between COVID-19-related stress and negative affect. In contrast, those with more vivid positive future imagery had weaker associations between COVID-19-related stress and anhedonia. These findings broadly support results by Pile and Lau (2018) which found that elevated vivid positive future imagery protected against the effects of life events on symptoms of depression in adolescents. A potential mechanism by which positive future mental imagery may reduce depressive symptoms particularly anhedonia is through its associations with optimism, psychological wellbeing and resilience to stress (Blackwell et al., 2013; Ji et al., 2017). Although these findings are intriguing, the cross-sectional nature of both our data and the data reported by Pile and Lau (2018) limits our ability to tease out whether these cognitive factors precede or follow symptoms of depression. Individuals with increased negative future imagery and reduced positive future imagery may be vulnerable to experiencing depression, but it is equally possible that depression symptoms impact the ability to generate vivid images.

We also gathered data to support the 2-factor structure of the Prospective Imagery Task and used this measure to explore the effects of demographic factors on positive and negative future images. Previous research has shown the importance of memory in our ability to imagine future events (Byrne et al., 2007; Schacter et al., 2007), and as adolescence represents a period when autobiographical memory develops (Fivush et al., 2011), we explored whether age is related to vividness of positive and negative future images. Positive future images were more vivid in younger adolescents than “emerging adults”, but there were no age differences for negative future imagery. Gulyás et al. (2022) recently found that vividness of mental imagery declines as a function of biological age, and here, we found that this was true for positive future imagery only. Our results suggest that promoting positive future mental imagery may be important in those approaching young adulthood—which is also a juncture associated with transitions—to further education or employment. While previous research in young people and adults shows that females have more vivid mental images than males (Campos & Sueiro, 1993; Gulyás et al., 2022; Isaac & Marks, 1994), we found that these differences depend on the valence of the images. Females reported less vivid imagery for positive future events and more vivid imagery for negative future events than males. Finally, our data revealed some small but potentially interesting differences across minority and majority ethnic and socioeconomic groups that may require further exploration. White participants had reduced vividness of positive future imagery compared to minority ethnic group participants, but there were no differences between these groups for vividness of negative future imagery. In terms of SES, participants whose parents did not have a degree (i.e., lower SES) had reduced vividness of both positive and negative future imagery, compared to participants whose parents did have a degree (higher SES).

Our findings are limited by the cross-sectional design of data collection but also by the use of a non-clinical sample without clinical measures of depression. However, putting these in the context of existing findings, could reveal some interesting implications for the treatment of adolescent depression. First, targeting negative future imagery could be important for the treatment of adolescent depression. In comparison to verbal techniques, prospective mental imagery has received less attention in depression, despite intrusive and distressing memories being common in depression (Wheatley & Hackmann, 2011). Imagery re-scripting, which involves patients focusing on the content of their intrusive images/memories and vividly imagining alternative, more positive outcomes (Hackmann, 1998), has shown promising results in reducing distress associated with memories in depressed adults (Brewin et al., 2009). A recent randomised controlled trial in adolescents found that compared to a control group, receiving nondirective supportive therapy, young people who received an imagery-based cognitive behavioural intervention involving memory specificity training and imagery rescripting had reduced symptoms of depression, greater memory specificity and greater abilities at imagining future events (Pile et al., 2021). Imagery re-scripting for future feared situations has recently been used to facilitate behavioural experiments in social anxiety and has been shown to reduce levels of anxiety and reduce the predicted probability and severity of the feared outcome (Landkroon et al., 2022). As our results, at least cross-sectionally, indicate that elevated negative future imagery and reduced positive future imagery are associated with negative affect, re-scripting negative future images for more alternative, positive future images could be a potential mechanism to treat adolescent depression, whereby distress and predicted probability for negative future events may be reduced, though this would need to be explicitly tested.

Second, current psychological interventions do not adequately address anhedonia or bolster positive affect, which may lower overall efficacy of treatments in all young people, or particularly in those with anhedonic symptoms. In adults, psychological interventions using positive future mental imagery have been used to target symptoms of depression with promising results. These interventions involve Episodic Future Thinking (EFT) Activities and Positive Imagery Cognitive Bias Modification (CBM). EFT activities aim to enhance detail and vividness of images for all future events but particularly positive events, by increasing anticipatory pleasure (Hallford et al., 2020). As anticipatory pleasure is associated with motivation (Engel et al., 2013) and psycho-social functioning (Foussias et al., 2011), enhancing positive affect when anticipating positive future events may be a promising mechanism to reduce anhedonia (Schubert et al., 2020). Positive imagery CBM targets mental imagery and interpretation by repeated practice of generating positive mental imagery resolutions to ambiguous training stimuli, to create more positive imagery biases to allow individuals to automatically imagine positive resolutions to novel everyday ambiguous information (Blackwell et al., 2015; Holmes et al., 2016). Depressed individuals who received positive imagery CBM showed significantly reduced symptoms of anhedonia compared to controls (Blackwell et al., 2015) and increased positive affect (Holmes et al., 2016), but not other symptoms of depression (Blackwell et al., 2015). Whilst these results are in adult populations, Burnett-Heyes et al. (2017) found that generating positive images increased positive affect and reduced negative interpretation bias in healthy adolescents.

Other than the cross-sectional design and use of a non-clinical sample, there are some additional limitations of the current study. First, the PIT replies on self-report, making it difficult to assess how accurate vividness ratings are. Moreover, when administering the PIT, we did not prompt for content, emotional intensity, or likelihood of the images. Vividness ratings could therefore be further confounded by differences in the likelihood of events and the emotional intensity of the events particularly where the items referred to more general categories of events. Additionally, it is possible that life events related to events on the PIT may have influenced vividness ratings, e.g. “You will be unwell” may be more vivid to a young person during the COVID-19 pandemic. Future studies using the PIT in young people should consider rating the emotional intensity of the images generated and the likelihood of each event. A final caveat around only using the PIT in this study is that the data cannot shed light on whether future thinking more generally is affected in those with high negative affect and anhedonia—or if it is prospective imagery specifically which correlates with affective disturbances. The addition of non-imagery-based measures of prospective cognition could identify unique and complementary effects of these aspects of future thinking. Second, we collected data during the COVID-19 pandemic. While this enabled us to assess the degree to which imagery variables may moderate the effects of stress on depression symptoms, it is difficult to know whether these will generalise to daily stressors and challenges beyond the pandemic. Third, a minority of our sample (n = 42) resided in countries outside of the UK. We assumed that most participants were comfortable with reading English (and therefore understanding questionnaire items) because the study was promoted and disseminated through UK-based educational institutions, charities and social media accounts. Nonetheless, as we did not formally assess reading comprehension, this may have affected the validity of questionnaire scores.

In closing, our results indicate that in adolescence, symptoms of negative affect are associated with having more vivid negative future imagery and less vivid positive future imagery, whereas symptoms of anhedonia are only associated with having reduced vividness of positive future imagery. Vividness of negative future imagery moderated the relationship between the impact of COVID-19 and negative affect, and vividness of positive future imagery moderated the relationship between the impact of COVID-19 and anhedonia, but not vice-versa. These findings build on previous literature exploring the role of prospective imagery in adolescent depression. With further replications in longitudinal designs and in clinical samples, they could inform novel mechanisms to improve psychological treatments for young people with depression.

## Supplementary Information

Below is the link to the electronic supplementary material.

Supplementary file1 (DOCX 156 KB)

## Data Availability

The data that support the findings of this study are openly available at: Hutchinson, Taryn (2022), “Is future mental imagery associated with reduced impact of the COVID-19 pandemic on negative affect and anhedonic symptoms in young people?”, Mendeley Data, V1, 10.17632/pprxpzvbcs.1.
