# Supplementary material for: Is Future Mental Imagery Associated with Reduced Impact of the COVID-19 Pandemic on Negative Affect and Anhedonic Symptoms in Young People?
Source: Cognit Ther Res. 2023 Feb 4;47(2):168–80. doi: 10.1007/s10608-023-10352-1 (PMC9899113; doi:10.1007/s10608-023-10352-1)
Supplement: Supplementary file 1 — Supplementary file1 (DOCX 156 KB) [file 10608_2023_10352_MOESM1_ESM.docx]

**Supplementary Materials**

**Table 1.** Number of participants by country

| **Country** | ***n*** |
| --- | --- |
| Bangladesh | 1 |
| Bulgaria | 1 |
| China | 3 |
| Czech Republic | 1 |
| France | 4 |
| Greece | 1 |
| India | 6 |
| Italy | 1 |
| Jordan | 1 |
| Kenya | 1 |
| Malaysia | 1 |
| Netherlands | 1 |
| Pakistan | 2 |
| Poland | 2 |
| Portugal | 1 |
| Russia | 2 |
| Singapore | 1 |
| South Africa | 2 |
| Spain | 2 |
| Switzerland | 1 |
| Turkey | 2 |
| United Kingdom | 2560 |
| United States of America | 5 |

**Table 2. Average length of time (in days) between surveys**

| Waves | Mean (SD) |
| --- | --- |
| 1-2 | 19.75 (13.82) |
| 2-3 | 17.50 (11.78) |
| 3-4 | 16.35 (8.70) |
| 4-5 | 17.77 (11.22) |
| 5-6 | 16.82 (8.85) |
| 6-7 | 15.61 (5.78) |
| 7-8 | 15.70 (6.39) |

**Snaith-Hamilton Pleasure Scale – Adapted for use with young people (5 items removed)**

This questionnaire is designed to measure your ability to experience pleasure IN THE LAST 2 WEEKS. Circle one of the boxes to indicate how much you agree or disagree with each statement.

Response options: Strong disagree, disagree, agree, strongly agree

a) I would enjoy my favourite television or radio programme

b) I would find pleasure in my hobbies and pastimes

c) I would enjoy my favourite meal

d) I would enjoy a warm bath or refreshing shower

e) I would enjoy seeing other people’s smiling faces

f) I would enjoy reading a book, magazine or newspaper

g) I would enjoy a cup of tea or coffee or my favourite drink

h) I would get pleasure from helping others

i) I would feel pleasure when I receive praise from other people


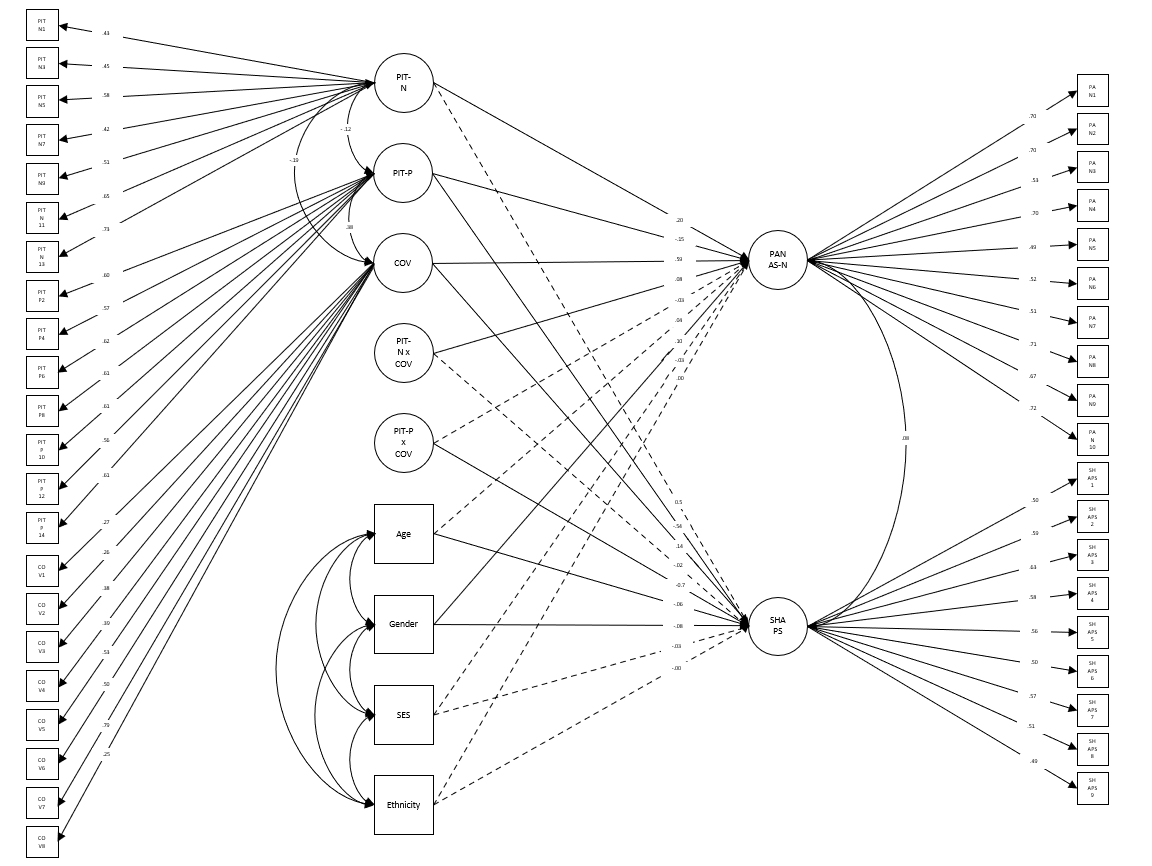


**Fig 1.** Full moderation SEM model denoting all estimated pathways.
*Note.* Standardised beta coefficients and standard errors are reported. Solid lines indicate significant associations at *p <* .05. Path coefficients are also reported in Table 3. Factor loadings as estimated in Fig 1. are reported in Table 4.

**Table 3.** Path coefficients

| Relationship | | Estimate (β) | SE | *P* |
| --- | --- | --- | --- | --- |
| PANAS-N | PIT-N  PIT-P  COVID  PIT-N X COVID  PIT-P X COVID  AGE  SEX  SES  ETHNICITY | .20 | .03 | <.001 |
|  |  | -.15 | .02 | <.001 |
|  |  | .59 | .02 | <.001 |
|  |  | .08 | .02 | <.001 |
|  |  | -.03 | .02 | .150 |
|  |  | .04 | .02 | .055 |
|  |  | .10 | .02 | <.001 |
|  |  | -.03 | .02 | .179 |
|  |  | .002 | .02 | .915 |
| SHAPS | PIT-N  PIT-P  COVID  PIT-N X COVID  PIT-P X COVID  AGE  SEX  SES  ETHNICITY | .05 | .03 | .108 |
|  |  | -.54 | .02 | <.001 |
|  |  | .14 | .03 | <.001 |
|  |  | -.02 | .03 | .497 |
|  |  | -.07 | .03 | .025 |
|  |  | -.06 | .02 | .012 |
|  |  | -.08 | .02 | <.001 |
|  |  | -.03 | .02 | .163 |
|  |  | .000 | .02 | .999 |

**Table 4.** Factor loadings

| Item | Loading |
| --- | --- |
| PIT-N1 | .43 |
| PIT-N3 | .45 |
| PIT-N5 | .58 |
| PIT-N7 | .42 |
| PIT-N9 | .51 |
| PIT-N11 | .65 |
| PIT-N13 | .73 |
| PIT-P2 | .60 |
| PIT-P4 | .57 |
| PIT-P6 | .62 |
| PIT-P8 | .61 |
| PIT-P10 | .61 |
| PIT-P12 | .56 |
| PIT-P14 | .61 |
| COVID1 | .27 |
| COVID2 | .26 |
| COVID3 | .38 |
| COVID4 | .39 |
| COVID5 | .53 |
| COVID6 | .50 |
| COVID7 | .79 |
| COVID8 | .25 |
| PANAS-N1 | .70 |
| PANAS-N2 | .70 |
| PANAS-N3 | .53 |
| PANAS-N4 | .70 |
| PANAS-N5 | .49 |
| PANAS-N6 | .52 |
| PANAS-N7 | .51 |
| PANAS-N8 | .71 |
| PANAS-N9 | .67 |
| PANAS-N10 | .72 |
| SHAPS1 | .50 |
| SHAPS2 | .59 |
| SHAPS3 | .63 |
| SHAPS4 | .58 |
| SHAPS5 | .56 |
| SHAPS6 | .50 |
| SHAPS7 | .57 |
| SHAPS8 | .51 |
| SHAPS9 | .49 |
